# Editorial: In the June 2017 issue

**DOI:** 10.1590/1980-57642016dn11-020001

**Published:** 2017

**Authors:** Ricardo Nitrini

**Affiliations:** Editor-in-Chief

In the June 2017 issue we are publishing one review, nine original papers, one history note and four case reports.

**Nazareth** reviewed the evidence linking diabetes mellitus type 2 (DM) with Alzheimer's disease, proposing that the inflammation which occurs in both diseases may be responsible for the increased risk of Alzheimer's disease in individuals with insulin resistance and DM.

**Carvalho et al.** carried out a systematic review to evaluate the knowledge on the influence of education on the performance of older adults in digital cognitive tests. Only a few papers were selected and there was no effect of education on performance. However, the authors reinforce the need for further studies, particularly including individuals with very low education.

**Conti** performed a systematic review to identify the available instruments for cognitive assessment by Occupational Therapists in clinical practice in Brazil. There were 27 unvalidated and 25 validated instruments in Brazilian Portuguese, but only one of the validated instruments was specific for Occupational Therapists.

**Musa et al.** evaluated the psychometric properties of the Spanish version of the Neuropsychiatric Inventory-Q (NPI-Q) in a sample of patients with Alzheimer's disease and their caregivers. They found that NPI-Q is a suitable instrument for the assessment of neuropsychological symptoms in Alzheimer's disease in Chile.

**Satler et al.** compared the planning ability of patients with Alzheimer's disease and elderly controls using the Tower of London test. The authors found that patients were less accurate and less efficient than controls due to working memory and inhibitory deficits. These findings may be relevant for rehabilitation programs.

**Malak et al.** investigated the depressive symptoms of patients with Parkinson disease and mild cognitive impairment using several cognitive tests and the Beck Depression Inventory. Their main conclusion was that symptoms of depression may be linked to Parkinson disease itself whereas other are related to cognitive impairment, and that the distinction is not simple but necessary.

**Silva et al.** evaluated the prevalence of sundown syndrome in the wards of a university hospital using the Confusion Assessment Method (CAM), and investigated its relationship with cognitive impairment and anxiety/depression symptoms. Age, cognitive impairment and depression were associated with a higher prevalence of delirium in this hospital setting.

**Borges et al.** performed an adaptation of the tool for evaluating risk of Alzheimer's disease - the "Australian National University - Alzheimer's Disease Risk Index" for use in Brazil. After following the standards of cross-cultural adaptation, the authors found the final version easily understandable by the Brazilian population.

**Ianof et al.** evaluated the electroencephalographic differences between Alzheimer's disease and diffuse axonal injury induced by trauma. eLoretta analysis revealed differences between the conditions and among them and a control group, but electrical activity was impaired in areas important for memory and learning for both pathologic conditions.

**Ordonez et al.** investigated the effects of training using an electronic game (Actively Station) on cognitive test performance in healthy elders. After 12 session of training, memory and language were the domains that showed most improvement. There was also a decline in an anxiety index when performance was compared with that of a control group.

**Engelhardt and Gomes** investigated an intriguing fact related to the history of the Lewy's body discover. Despite his continuous work on the neuropathology of diseases affecting the basal ganglia, Lewy did not mention his own discovery after 1923. The reasons for this neglect (or rejection) remain unknown.

**Cao et al.** reported a case of prolonged delirium diagnosed as a mood disorder, emphasizing the risks of misdiagnosis in these cases, where early adequate management can improve prognosis.

**João et al.** reported a case of a young man with known cardiac disease who presented with a stroke and transient Gerstmann Syndrome (GS), characterized by the association of agraphia, acalculia, right-left disorientation and finger agnosia. The possibility of disconnection syndrome is discussed.

**Figueiredo et al.** reported a case of catatonia as the initial manifestation of cerebral venous thrombosis, discussing the diagnosis of catatonia, its possible mechanisms in this case and the challenges establishing the diagnosis.

**Sousa** described a case with probable Korsakoff syndrome due to prolonged malnutrition, following several abdominal surgical procedures. After a rehabilitation program there was an improvement in level of recall with repetition of tasks.

**Ricardo Nitrini** Editor-in-Chief

